# Epigenetic Age Acceleration and Hearing: Observations From the Baltimore Longitudinal Study of Aging

**DOI:** 10.3389/fnagi.2021.790926

**Published:** 2021-12-15

**Authors:** Pei-Lun Kuo, Ann Zenobia Moore, Frank R. Lin, Luigi Ferrucci

**Affiliations:** ^1^Translational Gerontology Branch, National Institute on Aging, National Institutes of Health, Baltimore, MD, United States; ^2^Department of Otolaryngology–Head and Neck Surgery, Johns Hopkins University School of Medicine, Baltimore, MD, United States; ^3^Cochlear Center for Hearing and Public Health, Johns Hopkins University Bloomberg School of Public Health, Baltimore, MD, United States

**Keywords:** aging, age-related hearing loss (ARHL), epigenetic clock, DNA methylation, pace of aging, phenotypic aging, functional aging, epigenetic age acceleration

## Abstract

**Objectives:** Age-related hearing loss (ARHL) is highly prevalent among older adults, but the potential mechanisms and predictive markers for ARHL are lacking. Epigenetic age acceleration has been shown to be predictive of many age-associated diseases and mortality. However, the association between epigenetic age acceleration and hearing remains unknown. Our study aims to investigate the relationship between epigenetic age acceleration and audiometric hearing in the Baltimore Longitudinal Study of Aging (BLSA).

**Methods:** Participants with both DNA methylation and audiometric hearing measurements were included. The main independent variables are epigenetic age acceleration measures, including intrinsic epigenetic age acceleration—“IEAA,” Hannum age acceleration—“AgeAccelerationResidualHannum,” PhenoAge acceleration—“AgeAccelPheno,” GrimAge acceleration—“AgeAccelGrim,” and methylation-based pace of aging estimation—“DunedinPoAm.” The main dependent variable is speech-frequency pure tone average. Linear regression was used to assess the association between epigenetic age acceleration and hearing.

**Results:** Among the 236 participants (52.5% female), after adjusting for age, sex, race, time difference between measurements, cardiovascular factors, and smoking history, the effect sizes were 0.11 995% CI: (–0.00, 0.23), *p* = 0.054] for Hannum’s clock, 0.08 [95% CI: (–0.03, 0.19), *p* = 0.143] for Horvath’s clock, 0.10 [95% CI: (–0.01, 0.21), *p* = 0.089] for PhenoAge, 0.20 [95% CI: (0.06, 0.33), *p* = 0.004] for GrimAge, and 0.21 [95% CI: (0.09, 0.33), *p* = 0.001] for DunedinPoAm.

**Discussion:** The present study suggests that some epigenetic age acceleration measurements are associated with hearing. Future research is needed to study the potential subclinical cardiovascular causes of hearing and to investigate the longitudinal relationship between DNA methylation and hearing.

## Introduction

Age is one of the strongest risk factors for hearing loss which is prevalent in nearly two-thirds of adults over 70 years (Goman and Lin, 2016; Kuo et al., 2021). According to the geroscience paradigm, most age-related chronic diseases are caused by the shared biological mechanisms of aging (Ferrucci et al., 2018; Kuo et al., 2020). Among these hallmarks of aging, epigenetic change and DNA methylation have been associated with chronic disease (Salameh et al., 2020; Oblak et al., 2021). It is plausible that epigenetic mechanisms also contribute to hearing loss (Walters and Cox, 2019; Leso et al., 2020).

We have previously hypothesized that there is a hierarchical and temporal relationship between biological aging, phenotypic aging, and functional aging (Ferrucci et al., 2018; Kuo et al., 2020). Thus, biomarkers that capture the rate of biological aging may capture the early development of age-related diseases such as hearing loss prior to the full onset of symptoms and perception of deficits by the individual (Ferrucci et al., 2018; Kuo et al., 2020). Over the past decade, DNA methylation has emerged as one of the most promising biomarkers that captures biological aging (Oblak et al., 2021). Previous studies have demonstrated that independent of chronological age, DNA methylation clock algorithms predict several health outcomes, including chronic diseases and mortality (Oblak et al., 2021). There have been several recent refinements of methods first suggested by Bocklandt et al. (2011); Hannum et al. (2013), and Horvath (2013). Levine et al. (2018) developed an algorithm using an aggregate measure of phenotypic aging (“PhenoAge”) that included biomarkers that are commonly measured in a clinical setting trained by predicting mortality, and then train the epigenetic clock on this aggregate measure of phenotypic aging. Lu et al. (2019) used an alternate strategy first building epigenetics-based measures for informative aging markers and smoking pack-years, and then summarize these measures into a composite score (“GrimAge”). Belsky et al. (2020) proposed to first summarize the longitudinal rate of changes across several available phenotypes to then create a summarized epigenetic score (“DunedinPoAm”) to predict the summarized rate of changes.

Although the relationship between these epigenetic measures and many early life exposures as well as age-related functional decline (e.g., declining cognitive and physical function) have been widely studied (Oblak et al., 2021), the relationship between accelerated epigenetic measures and hearing has not been established yet. In this paper, we examine the relationship between several epigenetic clocks and audiometric hearing in the Baltimore Longitudinal Study of Aging (BLSA).

## Materials and Methods

### Study Population

The BLSA is a study of healthy aging conducted by the National Institute on Aging Intramural Research Program. Established in 1958 and comprehensively revised in 2003, the BLSA includes extensive domain-based phenotypic measurements and molecular biomarkers (Kuo et al., 2020). All participants are community-dwelling volunteers free of major chronic conditions upon enrollment. Detailed inclusion/exclusion criteria are described in our previous work (Kuo et al., 2020). DNA extracted from buffy coats derived from overnight fasting blood samples were used for DNA methylation measurement. The study protocol has been approved by the Internal Review Board of the Intramural Research Program of the National Institutes of Health and participants provided written informed consent at each visit. The study sample for the analyses described here includes participants with DNA methylation measurement and audiometric hearing measurements no more than 6 years apart.

### DNA Methylation and Epigenetic Age Acceleration

DNA methylation was assayed using DNA extracted from blood samples collected at visits between November 1993 and March 2010. CpG methylation status of 485,577 CpG sites was determined using the Illumina Infinium HumanMethylation450 BeadChip (Illumina Inc., San Diego, CA) per the manufacturer’s protocol. Data processing included NOOB and BMIQ normalization using R package “minfi” (Aryee et al., 2014). Multi-dimension scaling-defined outliers, as well as sex and SNP discordant samples were excluded in quality control. The following chronological age independent epigenetic measures were calculated: intrinsic epigenetic age acceleration---‘‘IEAA,’’ Hannum age acceleration---‘‘AgeAccelerationResidualHannum,’’ PhenoAge acceleration---‘‘AgeAccelPheno,’’ GrimAge acceleration---‘‘AgeAccelGrim,’’ and methylation-based pace of aging estimation ---‘‘ DunedinPoAm’’ (or ‘‘POAm_38_Dunedin’’ in this paper). Epigenetic ages were calculated using the Horvath online calculator^1^ or ‘‘projector’’ package.^2^ As to the construction of PhenoAge, Levine et al. (2018) “first” used the blood-based clinical phenotypes (albumin, creatinine, glucose, c-reactive protein, lymphocyte percentage, mean cell volume, red cell distribution with, alkaline phosphatase white blood cell count), and chronological age. To calculate the pace of aging in Dunedin cohort, Belsky et al. (2020) incorporated the longitudinal changes in 18 phenotypes (cardiorespiratory fitness, mean arterial pressure, lipoprotein, triglycerides, total cholesterol, HDL cholesterol, ApoB100/ApoA, and etc.). As to GrimAge, Lu et al. (2019) leveraged on the epigenetic estimates of seven proteins and smoke-pack years to generate the methylation-based GrimAge.

### Audiometry

Audiometric testing was conducted using a sound-attenuating booth and an Interacoustics AD629 audiometer with ER3A insert earphones by trained technicians. Air-conduction thresholds were assessed in each ear at octave frequencies from 0.5 to 8 kHz. The speech-frequency pure tone average (PTA) defined by thresholds at 0.5, 1, 2, and 4 kHz in the better ear was used in the main analyses (Lin et al., 2011). Higher PTA means worse hearing.

### Other Covariates

Age, sex, race, smoking history, and history of hypertension, diabetes, congestive heart failure, peripheral arterial disease were obtained from health history interviews and examinations conducted by the trained health professionals. As to detailed smoking history, participants were asked about their history of using pipe tobacco, cigarette, and cigar.

### Statistical Analysis

Linear regression was used to estimate the association between epigenetic measurements (independent variable) and audiometric hearing (dependent variable). In model 1, we adjusted for sex, black race, age, and time difference between epigenetic measurement and hearing measurement. In model 2, to account for potential confounding by cardiovascular diseases, we further adjusted for hypertension, diabetes, congestive heart failure, and peripheral arterial disease. Because smoking history was used as an intermediate step to create GrimAge, in model 3, to account for the potential confounding by smoking, we further adjusted for smoking history (4 category: never smoker, quit over 10 years ago, quit less than 10 years ago, and current smoker). Since not all epigenetic clocks were developed using populations with wide age ranges, we performed a subgroup analysis limiting to those aged 60 and above. Additional sensitivity analysis evaluating smoking using pack-years including pipe tobacco, cigarette, and cigar history were also conducted. Further, we also conducted the other sensitivity analysis using the PTA at worse ear as dependent variable. All standardized estimates were reported with 95% confidence interval (CI). The analysis was conducted using R 3.6.

## Results

Baseline characteristics of the analytic population are shown in Table 1. Of the 236 participants, 124 (52.5%) were female, and 135 (57.2%) had never smoked. The mean epigenetic ages across epigenetic measures were lower than the mean chronological age of the study sample: Hannum—67.81 (SD: 11.47), Horvath—66.65 (SD: 10.42), PhenoAge—59.39 (SD: 12.16), and GrimAge —63.69 (SD:9.72). The mean pace of aging estimated by DNA methylation was 1.02 (SD: 0.08).

**TABLE 1:** Baseline characteristics among 236 participants in Baltimore Longitudinal Study of Aging.

Characteristics	Overall	Female	Male
**Mean (SD)/n (%)**			
N	236 (100)	124 (52.5)	112 (47.5)
Age	68.39 (10.73)	67.24 (10.90)	69.67 (10.44)
Black	65 (27.5)	40 (32.3)	25 (22.3)
Pure tone average^a^	29.75 (14.98)	27.31 (14.80)	32.46 (14.77)
**Epigenetic summary^b^**			
Hannum’s DNAmAge	67.81 (11.47)	65.23 (11.44)	70.66 (10.85)
Horvath’s DNAmAge	66.65 (10.42)	65.15 (10.29)	68.33 (10.35)
DNAmPhenoAge	59.39 (12.16)	57.90 (12.21)	61.03 (11.94)
DNAmGrimAge	63.69 (9.72)	61.12 (9.52)	66.54 (9.16)
DunedinPoAm	1.02 (0.08)	1.00 (0.08)	1.04 (0.08)
**Other covariates**			
Hypertension	154 (73.3)	71 (65.1)	83 (82.2)
Diabetes	38 (18.1)	11 (10.1)	27 (26.7)
Congestive heart failure	35 (16.7)	13 (11.9)	22 (21.8)
Peripheral arterial disease	7 (3.3)	2 (1.8)	5 (5.0)
**Smoke history**			
Never	135 (57.2)	77 (62.1)	58 (51.8)
Quit over 10 years ago	93 (39.4)	45 (36.3)	48 (42.9)
Quit less than 10 years ago	4 (1.7)	1 (0.8)	3 (2.7)
Current	4 (1.7)	1 (0.8)	3 (2.7)

*^a^Pure tone average was defined as average thresholds across speech frequency (0.5–4 Hz). Higher pure tone average means worse hearing.*

*^b^Epigenetic Summary includes Hannum’s DNAmAge (the clock developed by Hannum et al., 2013), Horvath’s DNAmAge (the clock developed by Horvath, 2013), DNAmPhenoAge (the clock developed by Levine et al., 2018), DNAmGrimAge (the clock developed by Lu et al., 2019), and DunedinPoAm (the clock developed by Belsky et al., 2020).*

**FIGURE 1:**
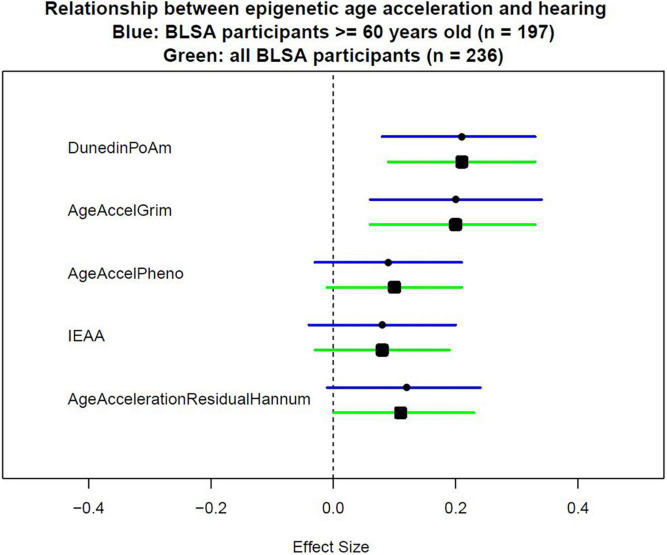
Effect sizes of epigenetic measurements on hearing. To measure epigenetic age acceleration, the chronological age-adjusted version was used. AgeAccelerationResidualHannum is the chronological age-adjusted version for the epigenetic clock proposed by Hannum et al. (2013). IEAA is the chronological age-adjusted version for the epigenetic clock proposed by Horvath (2013). AgeAccelPheno is the chronological age-adjusted version for the epigenetic clock proposed by Levine et al. (2018). AgeAccelGrim is the chronological age-adjusted version for the epigenetic clock proposed by Lu et al. (2019). DunedinPoAm is the epigenetic score proposed by Belsky et al. (2020) which did not need additional adjustment for chronological age.

Because not all epigenetic age acceleration measurements are with the same unit, we quantify the association using effect size that corresponds to the standard deviation difference in PTA associated with a one standard deviation increase in epigenetic age acceleration. As shown in Table 2, after adjusting for age, sex, race, and measurement time interval, the association between all epigenetic measures and hearing was positive with statistically significant associations for GrimAge (“AgeAccelGrim”) with effect size of 0.19 (*p* = 0.001), and pace of aging estimate (“POAm_38_Dunedin”) with effect size of 0.19 (*p* < 0.001) (Table 2, Model 1). The direction and magnitude of associations were consistent after additional adjustment for hypertension, diabetes, congestive heart failure, peripheral arterial disease, and smoking history (Table 2, Model 3, and Figure 1).

**TABLE 2:** Association between summarized epigenetic measurements and hearing (*n* = 236).

	Model 1			Model 2			Model 3		
Epigenetic measurement^a^	Estimates^b^	95% CI	*p*-value	Estimates^b^	95% CI	*p*-value	Estimates^b^	95% CI	*p*-value
AgeAccelerationResidualHannum	0.07	–0.03–0.18	0.159	0.10	–0.02–0.21	0.090	0.11	–0.00–0.23	0.054
IEAA	0.08	–0.02–0.18	0.125	0.07	–0.04–0.18	0.204	0.08	–0.03–0.19	0.143
AgeAccelPheno	0.08	–0.02–0.18	0.104	0.09	–0.02–0.20	0.108	0.10	–0.01–0.21	0.089
AgeAccelGrim	0.19	0.08–0.31	**0.001**	0.21	0.08–0.33	**0.001**	0.20	0.06–0.33	**0.004**
DunedinPoAm	0.19	0.09–0.30	**< 0.001**	0.21	0.10–0.33	**< 0.001**	0.21	0.09–0.33	**0.001**

*Linear regression was used for calculating the association between epigenetic measurements and hearing. Speech-frequency pure tone average at better ear was used for hearing measurement, and treated as the dependent variable in the linear regression.*

*Model 1: adjusted for sex, black, age, time difference between epigenetic and hearing measurements.*

*Model 2: adjusted for sex, black, age, time difference between epigenetic and hearing measurements, hypertension, diabetes, congestive heart failure, and peripheral arterial disease.*

*Model 3: adjusted for sex, black, age, time difference between epigenetic and hearing measurements, hypertension, diabetes, congestive heart failure, peripheral arterial disease, and smoke history.*

*^a^To measure epigenetic age acceleration, the chronological age-adjusted version was used. AgeAccelerationResidualHannum is the chronological age-adjusted version for the epigenetic clock proposed by Hannum et al. (2013). IEAA is the chronological age-adjusted version for the epigenetic clock proposed by Horvath (2013). AgeAccelPheno is the chronological age-adjusted version for the epigenetic clock proposed by Levine et al. (2018). AgeAccelGrim is the chronological age-adjusted version for the epigenetic clock proposed by Lu et al. (2019). DunedinPoAm is the epigenetic score proposed by Belsky et al. (2020) which did not need additional adjustment for chronological age.*

*^b^Estimates referred to the estimate of effect size. Bold indicates p < 0.05.*

Because some epigenetic clocks were developed in older populations, a subgroup analysis limiting to 197 participants aged 60 and above was performed. Models were estimated with the same covariates as the analyses performed in the full study sample. Similar direction and magnitude of association were observed in the subset of older participants with statistically significant associations observed for GrimAge (“AgeAccelGrim”) 0.20 (*p* = 0.005), and pace of aging estimate (“POAm_38_Dunedin”) 0.21 (*p* = 0.001) in the fully adjusted model (Table 3, Model 3, and Figure 1).

**TABLE 3:** Association between summarized epigenetic measurements and hearing among those aged 60 years and above (*n* = 197).

	Model 1			Model 2			Model 3		
Epigenetic measurement^a^	Estimates^b^	95% CI	*p*-value	Estimates^b^	95% CI	*p*-value	Estimates ^b^	95% CI	*p*-value
AgeAccelerationResidualHannum	0.07	–0.05–0.19	0.242	0.10	–0.02–0.22	0.119	0.12	–0.01–0.24	0.066
IEAA	0.07	–0.04–0.19	0.211	0.07	–0.05–0.18	0.277	0.08	–0.04–0.20	0.192
AgeAccelPheno	0.08	–0.04–0.20	0.194	0.08	–0.04–0.20	0.183	0.09	–0.03–0.21	0.150
AgeAccelGrim	0.21	0.08–0.34	**0.001**	0.22	0.09–0.35	**0.001**	0.20	0.06–0.34	**0.005**
DunedinPoAm	0.19	0.07–0.31	**0.002**	0.22	0.10–0.34	**< 0.001**	0.21	0.08–0.33	**0.001**

*Linear regression was used for calculating the association between epigenetic measurements and hearing. Speech-frequency pure tone average at better ear was used for hearing measurement, and treated as the dependent variable in the linear regression.*

*Model 1: adjusted for sex, black, age, time difference between epigenetic and hearing measurements.*

*Model 2: adjusted for sex, black, age, time difference between epigenetic and hearing measurements, hypertension, diabetes, congestive heart failure, and peripheral arterial disease.*

*Model 3: adjusted for sex, black, age, time difference between epigenetic and hearing measurements, hypertension, diabetes, congestive heart failure, peripheral arterial disease, and smoke history.*

*^a^To measure epigenetic age acceleration, the chronological age-adjusted version was used. AgeAccelerationResidualHannum is the chronological age-adjusted version for the epigenetic clock proposed by Hannum et al. (2013) IEAA is the chronological age-adjusted version for the epigenetic clock proposed by Horvath (2013) AgeAccelPheno is the chronological age-adjusted version for the epigenetic clock proposed by Levine et al. (2018) AgeAccelGrim is the chronological age-adjusted version for the epigenetic clock proposed by Lu et al. (2019) DunedinPoAm is the epigenetic score proposed by Belsky et al. (2020) which did not need additional adjustment for chronological age.*

*^b^Estimates referred to the estimate of effect size. Bold indicates p < 0.05.*

For the fine adjustment of smoking using pack-years, the results remained consistent (Supplementary Table 1). When the dependent variable changed from PTA at better ear to PTA at worse ear, the results remained consistent (Supplementary Figure 1).

## Discussion

To the best of our knowledge, this is the first study investigating the relationship between epigenetic clocks and hearing loss using objective audiometric measurement among healthy adults in the U.S. Our results demonstrate that the relationship between epigenetic clocks and hearing varies across epigenetic clock algorithms. In comparison to the Hannum clock, Horvath clock, and PhenoAge, GrimAge and pace of aging epigenetic clock have stronger associations with hearing, in terms of both magnitude and statistical significance.

Our results suggest that not all commonly cited epigenetic clocks may correlate with sensory function. Although these clocks have been shown to be associated with mortality, as well as physical and cognitive function, they may not capture the same aspects of aging (Oblak et al., 2021). The epigenetic clocks were constructed using different phenotype information in populations of varying ages. Previously it has been observed that the correlation between clocks is not always strong. Indeed, among BLSA participants who are typically healthier than general population, we observe younger epigenetic age (Hannum clock, Horvath clock, GrimAge, and PhenoAge) but the mean epigenetic measurement of pace of aging is 1.02, a value consistent with slightly accelerated aging. One potential explanation is that many age-related functional and phenotypic changes in fact accelerates at mid-to-late life, which the pace of aging may be unable to acknowledge because this epigenetic measure is established using only young adults (Belsky et al., 2020).

Despite the difference in how epigenetic clocks were built, two epigenetic measurements (GrimAge and epigenetic measurement of pace of aging) were associated with hearing. One potential explanation is that these two epigenetic clocks contain richer information than the other three clocks. The construction of GrimAge uses more biomarkers and smoking history in comparison to the other three epigenetic clocks (Hannum clock, Horvath clock, PhenoAge), and mortality information was directly used for training in the last step (Lu et al., 2019). Pace of aging is the only epigenetic measure that utilizes longitudinal trends of phenotypes in its construction (Belsky et al., 2020). Consequently, GrimAge and pace of aging may capture deviations from healthy aging operationalized as accelerated epigenetic age better than the other clocks.

Alternatively, our findings may be an indication of the impact of cardiovascular factors on hearing loss. Wattamwar et al. (2018) showed that cardiovascular comorbidities were associated with worse hearing, and in comparison to other cardiovascular factors, coronary artery disease has the strongest association with hearing. The authors attributed their finding to coronary artery disease being an indicator of cochlear microvascular disease (Wattamwar et al., 2018). Thus, it is reasonable to hypothesize that many subclinical cardiovascular changes that cannot be captured by the clinical diagnoses of cardiovascular disease may also affect cochlear microvascular environment and hearing. While adjusting for overt cardiovascular diseases and smoking did not substantially alter the associations between epigenetic clocks and hearing, the presence of association for GrimAge and pace of aging may reflect the dependence of their construction on cardiovascular measurements. GrimAge and pace of aging may be able to capture the cardiovascular aging that is developed before cardiovascular diseases are diagnosed (Hillary et al., 2020; Oblak et al., 2021), leading to the observed association with hearing.

The absence of association between hearing and several epigenetic clocks suggests both that the meaning of epigenetic clocks is highly dependent on the ways they are constructed (Oblak et al., 2021) and that there may be some unique biological pathways leading to age-related hearing deterioration that are not shared with other age-related functional decline (e.g., cognitive or physical function) (Oblak et al., 2021). Current epigenetic clocks capture only limited information on the factors that lead to age-related hearing loss (ARHL). To discover potential epigenetic influences on hearing, an epigenetic signature of hearing needs to be investigated separately.

We acknowledge several limitations to our study. First, the hearing and DNA methylation were not measured at the same time. Second, the DNA methylation was sampled from blood, which may not reflect the DNA methylation in cells of the inner ear or other tissues that are involved in the pathogenesis of hearing loss. Third, although DNA methylation was measured before hearing, since this is cross-sectional study, the results may be driven by unmeasured confounders, such as environmental/occupational noise exposure, and detailed medication history. Although the relationship between noise exposure and epigenetic age acceleration remains unclear, the observed association between epigenetic age acceleration and hearing is expected to be weakened if there is a strong association between noise exposure and epigenetic age acceleration. Fourth, because BLSA aims to study healthy aging, the participants in BLSA are healthier than the general population. Future studies are needed to understand whether the association between epigenetic age acceleration and hearing is stronger in the population with higher proportion of participants experiencing accelerated epigenetic aging. However, despite these limitations, the BLSA is one of the very few studies collecting both DNA methylation data and performing audiometric hearing testing. We believe these measurements analyzed in the context of a deeply phenotyped cohort represent an important first step in understanding biological aging processes and hearing loss.

## Conclusion

In conclusion, our findings demonstrate that not all epigenetic clocks were strongly correlated with hearing. Only those epigenetic clocks established using many cardiovascular measurements with longitudinal information were associated with hearing. Future research is needed to study the potential subclinical cardiovascular causes of hearing and to investigate the relationship between DNA methylation and hearing longitudinally.

## Data Availability Statement

The data analyzed in this study is subject to the following licenses/restrictions: BLSA data are available upon request *via* website portal (https://www.blsa.nih.gov/how-apply). Requests to access these datasets should be directed to https://www.blsa.nih.gov/how-apply.

## Ethics Statement

The studies involving human participants were reviewed and approved by the Internal Review Board of the Intramural Research Program of the National Institutes of Health. The patients/participants provided their written informed consent to participate in this study.

## Author Contributions

P-LK, AM, FL, and LF contributed to conception, design of the study, collected and organized the data, designed the analysis, interpreted the results of the statistical analysis, contributed to manuscript revision, read, and approved the submitted version. P-LK wrote the first draft of the manuscript. All authors contributed to the article and approved the submitted version.

## Conflict of Interest

FL reports being a consultant to Frequency Therapeutics, receiving speaker honoraria from Caption Call, and being the director of a public health research center funded in part by a philanthropic donation from Cochlear Ltd., to the Johns Hopkins Bloomberg School of Public Health. The remaining authors declare that the research was conducted in the absence of any commercial or financial relationships that could be construed as a potential conflict of interest. The handling editor declared a past co-authorship with one of the authors LF.

## Publisher’s Note

All claims expressed in this article are solely those of the authors and do not necessarily represent those of their affiliated organizations, or those of the publisher, the editors and the reviewers. Any product that may be evaluated in this article, or claim that may be made by its manufacturer, is not guaranteed or endorsed by the publisher.

## Footnotes

1
https://dnamage.genetics.ucla.edu/

2
https://github.com/danbelsky/DunedinPoAm38
