# Supplementary material for: Epigenetic Age Acceleration and Hearing: Observations From the Baltimore Longitudinal Study of Aging
Source: Front Aging Neurosci. 2021 Dec 15;13:790926. doi: 10.3389/fnagi.2021.790926 (PMC8714776; doi:10.3389/fnagi.2021.790926)
Supplement: Supplementary file 1 [file Table_1.pdf]

Supplemental Table 1. Association between summarized epigenetic measurements and hearing among those aged 60 years and above

	Model 4		
Epigenetic Measurement [a]	Estimates [b]	95% CI	p-value
AgeAccelerationResidualHannum	0.08	-0.04 – 0.21	0.172
IEAA	0.07	-0.05 – 0.18	0.277
AgeAccelPheno	0.05	-0.07 – 0.18	0.385
AgeAccelGrim	0.18	0.04 – 0.33	**0.011**
DunedinPoAm	0.20	0.07 – 0.33	**0.003**

Linear regression was used for calculating the association between epigenetic measurements and hearing. Speech-frequency pure tone average at better ear was used for hearing measurement, and treated as the dependent variable in the linear regression.
Model 4: adjusted for sex, black, age, time difference between epigenetic and hearing measurements, hypertension, diabetes, congestive heart failure, peripheral arterial disease, and smoke (pack-years)

[a] To measure epigenetic age acceleration, the chronological age-adjusted version was used. AgeAccelerationResidualHannum is the chronological age-adjusted version for the epigenetic clock proposed by Hannum et al. IEAA is the chronological age-adjusted version for the epigenetic clock proposed by Horvath et al. AgeAccelPheno is the chronological age-adjusted version for the epigenetic clock proposed by Levine et al. AgeAccelGrim is the chronological age-adjusted version for the epigenetic clock proposed by Lu et al. DunedinPoAm is the epigenetic score proposed by Belsky et al., which did not need additional adjustment for chronological age.
[b] Estimates referred to the estimate of effect size.

Supplemental Figure 1. Effect sizes of epigenetic measurements on hearing using worse ear

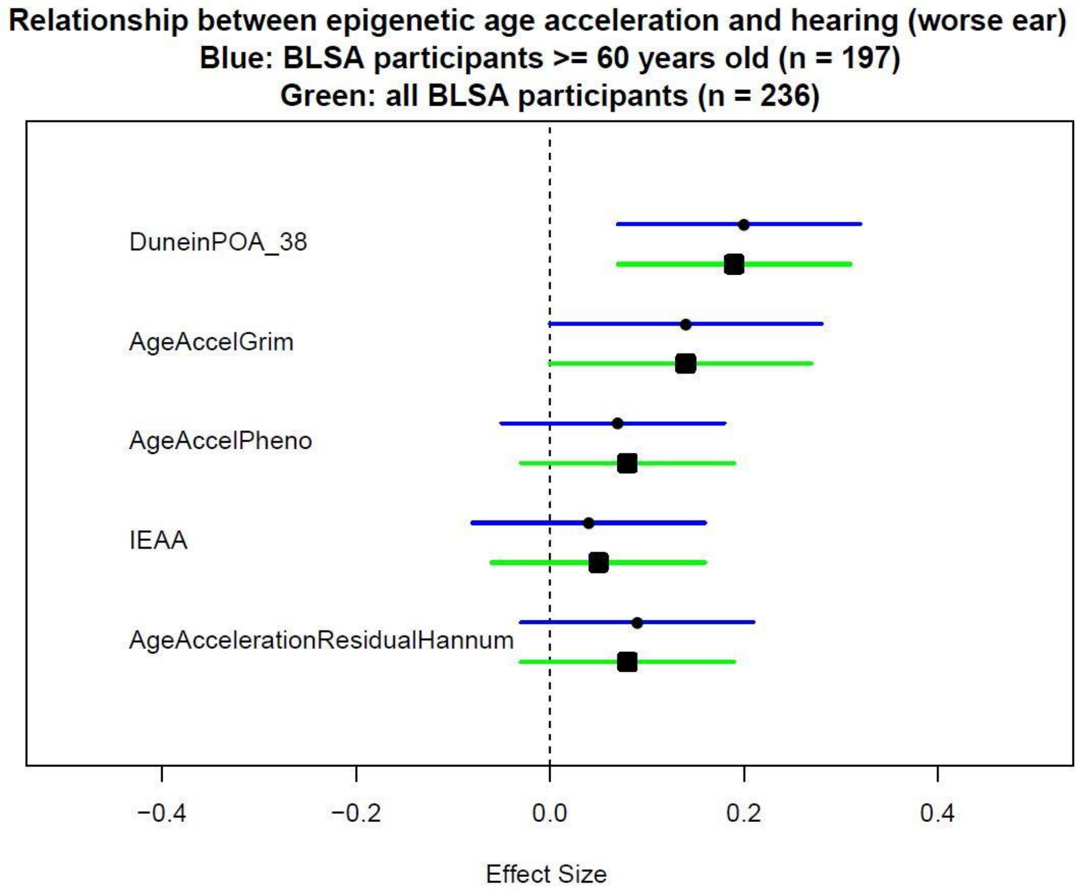

For this sensitivity analysis, the dependent variable is speech-frequency PTA at the worse ear instead of better ear. To measure epigenetic age acceleration, the chronological age-adjusted version was used. AgeAccelerationResidualHannum is the chronological age-adjusted version for the epigenetic clock proposed by Hannum et al. IEAA is the chronological age-adjusted version for the epigenetic clock proposed by Horvath et al. AgeAccelPheno is the chronological age-adjusted version for the epigenetic clock proposed by Levine et al. AgeAccelGrim is the chronological age-adjusted version for the epigenetic clock proposed by Lu et al. DunedinPoAm is the epigenetic score proposed by Belsky et al., which did not need additional adjustment for chronological age.
